# Clinical features of HAdV-55 in children with respiratory tract infections: a retrospective case series and literature review

**DOI:** 10.1186/s12879-025-10890-x

**Published:** 2025-04-17

**Authors:** Lifen Rao, Yueqiang Fu, Ying Lu, Jianhua Wei, Zhongying Yang, Mengling Qi, Chengjun Liu, Yushun Wan, Enmei Liu, Na Zang

**Affiliations:** 1 Department of Respiratory Children’s Hospital of Chongqing Medical University, National Clinical Research Center for Child Health and Disorders, Ministry of Education Key Laboratory of Child Development and Disorders. China International Science and Technology Cooperation base of Child development and Critical Disorders, Chongqing Key Laboratory of Child Rare Diseases in Infection and Immunity, Chongqing, 400014 China; 2 Department of Pediatric Intensive Care Unit, Children’s Hospital of Chongging Medical University, 400014 Chongqing, China; 3 College of Basic Medicine, Chongqing Medical University, Chongqing, 400016 China

**Keywords:** HAdV-55, Children, Infection, Clinical features, ARDS

## Abstract

**Background:**

Human adenovirus type 55 (HAdV-55) can lead to acute respiratory diseases, significant morbidity, and mortality in children.

**Methods:**

Hospitalized children diagnosed with HAdV-55 between September 2016 and March 2024 at the Children’s Hospital of Chongqing Medical University were retrospectively analyzed. HAdV-55 was detected through polymerase chain reaction and sequencing. Clinical data were collected, including demographic characteristics, clinical manifestations, laboratory findings, imaging results, treatment history, and prognosis. A literature search was conducted using the PubMed database and China National Knowledge Infrastructure from their inception to June 2024. Search terms included “HAdV-55”, “HAdV-11a”, “adenovirus type 55” and their derivatives. Clinical features were evaluated in conjunction with literature on HAdV-55 infections in children.

**Results:**

Five children with HAdV-55 infection were identified, including one mild, two severe, and two critical. The two critical patients exhibited progressive declines in total blood cell counts, hemoglobin levels and serum albumin levels within a short period. Adenoviral DNA was detected in pleural fluid or serum for them. They received mechanical ventilation, intravenous immunoglobulin, Methylprednisolone, blood transfusions, and antibiotics, while died for acute respiratory distress syndrome (ARDS). The remaining ones recovered and were discharged with good prognosis. A review of 56 cases, including those from this study, revealed that 61.9% (26/42) of infections were classified as severe or critical, with a mortality rate of 16.4% (9/55). Sequelae included bronchiolitis obliterans and bronchiectasis.

**Conclusions:**

The genetic inheritance of HAdV-55 remained stable, with an upward trend of HAdV-55 severe infection among children from 2000 to 2019. Early clinical symptoms of HAdV-55 infection were overlapped with other respiratory viral infections. Rapid declines in blood cell counts, hemoglobin levels and serum albumin, along with dynamic monitoring of viral loads in sterile fluids, may serve as prognostic indicators.

**Supplementary Information:**

The online version contains supplementary material available at 10.1186/s12879-025-10890-x.

## Background

Human adenovirus (HAdV) is a significant etiological agent of acute respiratory tract infections in children, with an overall detection rate of 13.0% among such cases [1, 2]. The prevalence is particularly pronounced in children aged 3 to 6 years, where the detection rate rises to 18.7% [1]. Although most HAdV infections are self-limiting, severe cases, such as HAdV pneumonia or disseminated disease, can result in mortality rates exceeding 50% if left untreated [3].

HAdV is classified into seven subgenera (HAdV-A through HAdV-G), comprising over 100 genotypes and 52 serotypes identified by the HAdV Working Group [4]. HAdV-55, a recombinant serotype, possesses an HAdV-14 backbone with partial HAdV-11 sequences in the hexon gene, giving rise to this novel serotype [5]. Due to technological limitations in earlier studies, this serotype was previously classified as “HAdV-11a [5]. First identified in 1969 during a large acute respiratory tract infection outbreak among Spanish military trainees, subsequent outbreaks occurred in Turkey (2004) and Singapore military trainees (2005) and Chinese senior high school students (2006) [6–9]. While these outbreaks were characterized by rapid transmission, mortality was relatively rare.

Over time, HAdV-55 has become more prevalent in the general population, causing severe disease and fatalities even in immunocompetent adults [10, 11]. Although HAdV-55 infections are uncommon in children, accounting for only 3.1–6.9% of acute respiratory tract infections in this population, the virus remains capable of causing severe or life-threatening pneumonia [12–19]. Despite its significance, comprehensive clinical characterization of HAdV-55 infections in children remains limited.

To address this gap, we analyzed five cases of HAdV-55-induced acute respiratory tract infections in children hospitalized between September 2016 and March 2024. Clinical manifestations and disease outcomes were documented, and a literature review of previously reported cases was conducted to provide a more detailed characterization of HAdV-55 acute respiratory tract infections in pediatric populations.

## Methods

### Study population

Pediatric patients diagnosed with HAdV-55 respiratory tract infections at the Children’s Hospital of Chongqing Medical University were retrospectively enrolled in the study, covering the period September 2016 to March 2024.

### Clinical data collection

Clinical data were collected, including demographic information, clinical symptoms, vital signs, laboratory and imaging studies, therapy, outcomes and complications. Severe pneumonia was defined as respiratory distress accompanied by hypoxemia (sustained saturation of peripheral oxygen (SpO2) < 90% at sea level) [20]. Children considered as having critical pneumonia had ≥ 1 of the major or ≥ 2 of the minor following criteria [20]: (1) Major criteria: invasive mechanical ventilation; fluid refractory shock; acute need for non-invasive positive pressure ventilation; hypoxemia requiring fraction of inspired oxygen (FiO2) greater than the inspired concentration or flow feasible in general care area; (2) Minor criteria: respiratory rate greater than the World Health Organization normal classification for age; apnea; increased work of breathing (e.g., retractions, dyspnea, nasal flaring, and grunting), PaO2/FiO2 ratio < 250, multilobar infiltrates, pediatric early warning score > 6, altered mental status, hypotension, presence of effusion, comorbid conditions (e.g., hemoglobin SS disease, immunosuppression, and immunodeficiency); and unexplained metabolic acidosis.

### Virus Preparation and viral DNA extraction

The HAdV-55 CQ2403 strain was isolated from Nasopharyngeal aspirates (NPAs) of case 5. The virus was cultured and amplified in A549 cells using a medium containing 2% fetal bovine serum. Viral DNA was extracted from the infected cultures using the QIAamp MinElute Virus Spin Kit (QIAGEN, Cat# 57704) according to the manufacturer’s provided instructions. Extracted DNA was dissolved in DEPC-treated water and submitted to BGI Genomics for next-generation sequencing.

### Sample collection and molecular typing using PCR

NPAs were collected from hospitalized pediatric inpatients diagnosed with respiratory infections. The samples were stored at 2–8 °C in viral transport medium immediately after collection, transported on ice, and subsequently preserved at − 80 °C until analysis. Viral genomic DNA was extracted using a QIAamp MinElute Virus Spin Kit and detected with TaqMan Universal Mister Mix (Applied Biosystems, Cat#: 4440040). DNA from samples testing positive by PCR was sent to BGI Genomics for sequencing. Molecular characterization focused on the final 300 bp region of the hexon gene, enabling differentiation among HAdV isolates. Sequencing results were cross-referenced with the NCBI database to identify HAdV types.

### Phylogenetic analysis

Phylogenetic relationships were analyzed using Molecular Evolutionary Genetics Analysis v11, as previously described [21]. Hylogenetic trees were constructed with 1 000 bootstrap replicates to ensure reliability.

### Literature search and data extraction

A literature search was conducted using the PubMed database and China National Knowledge Infrastructure. Search terms included all child (0–18 years) and “HAdV-55 or HAdV-11a” and their derivatives. Articles published in Chinese and English from the inception of the databases until June 2024 were considered. Extracted data included country or region of origin, sex, age, season of admission, clinical manifestations, signs, etiological findings, imaging results, diagnoses, treatments, complications, and outcomes. Exclusion criteria were: (1) literature reviews, letters to the editor, or opinion papers were excluded; (2)cases aged ≥ 18 years old; (2) pediatric literature with missing critical data.

### Risk of bias assessment

Two reviewers (LFR and YQF) independently assessed the risk of bias for the included studies. The Joanna Briggs Institute (JBI) Critical Appraisal Tools were applied, with specific checklists selected according to study design types [22].

### Statistical analysis

Statistical analysis and visualization were conducted using R version 4.2.1. In the descriptive analysis, continuous variables are presented as medians (IQR), and categorical variables are presented as frequencies (percentages). In the analytical section, continuous variables are analyzed using the Wilcoxon rank-sum test, while categorical variables are analyzed using Fisher’s exact test. This study conducts map-based analysis and visualization using the Idbview data source [23].

## Results

### Demographic characteristics

From September 2016 to March 2024, a total of 4,318 NPA samples were collected at the Children’s Hospital of Chongqing Medical University for pathogen screening. Among these, 168 samples tested positive for human adenovirus (HAdV). Genotyping of the HAdV-positive samples identified serotypes including HAdV-1, HAdV-2, HAdV-3, HAdV-4, HAdV-7, HAdV-14, and HAdV-55, with 5 cases confirmed as HAdV-55. The median age of these five patients were 21 months, ranging from 5 to 57 months, having no underlying conditions. The male-to-female ratio was 4:1. Seasonal distribution showed one case in spring, three in summer, and one in autumn (Table 1).

**Table 1:** Clinical data of five children with HAdV-55 infection in the Children’s Hospital of Chongqing Medical University

Cases	Age (months)	Sex	Admission time	Chief complaint	Length of hospital stay (days)	Co-detection	Primary diagnosis	Treatment	Complications	Outcome	Sequelae
Case 1	5	Male	2016/9/16	Fever 3 days, cough 2 days, wheezing, shortness of breath 1 day	3	*Staphylococcus aureus*	Severe pneumonia	Symptomatic supportive therapy	Respiratory failure, symptomatic diarrhea, liver impairment	Survived	None
Case 2	22	Male	2018/7/1	Fever 11 days	5	EBV, CMV	Bronchitis	Symptomatic supportive therapy	None	Survived	None
Case 3	57	Male	2019/7/21	Cough 1 month, aggravated with fever 5 days	9	*Streptococcus pneumoniae*, *Haemophilus influenzae*	Severe pneumonia	IVIG, Imipenem, and CPAP	Septicemia, respiratory failure, pleural effusion, pleurisy, hepatic impairment	Survived	None
Case 4	9	Female	2019/8/2	Cough more than 1 month, aggravated with intermittent fever 1 week	12	*Streptococcus pneumoniae*, syphilis suspected	Critical pneumonia	Methylprednisolone, IVIG, human albumin, erythrocyte suspension, cefazoxime, and IMV	Respiratory failure, toxic encephalopathy? Brain herniation, intracranial hemorrhage? Cerebral infarction? Pleural effusion, symptomatic diarrhea, coagulation disorders, liver impairment	Died	
Case 5	12	Male	2024/3/11	Cough more than 1 month with fever 6 days	4	None	Critical pneumonia	Methylprednisolone, IVIG, human albumin, erythrocyte suspension, Cefoperazone sulbactam sodium and IMV	ARDS, septicemia, pleural effusion, hepatic impairment	Died	

EBV, Epstein-Barr virus; CMV, cytomegalovirus; IVIG, intravenous immunoglobulin; CPAP, continuous positive airway pressure; IMV, invasive mechanical ventilation.

^a^,Human albumin was administered to correct hypoalbuminemia.

^b^, erythrocyte suspension was utilized to manage anemia.

### Clinical and laboratory characteristics

All patients presented with cough and high-grade fever, overlapping with other respiratory viral infections, additional symptoms included rhinorrhea, diarrhea, wheezing and sore throat. Upon hospital admission, three patients demonstrated moist crackles on lung auscultation. Four patients co-detected with other pathogens. Based on clinical criteria, one patient was diagnosed with mild respiratory infection, two severe pneumonia, and two critical pneumonia (Table 1). Two critical pneumonia, patient 4 and 5, exhibited significant short-term reductions in whole blood cell counts, hemoglobin levels and plasma albumin levels, accompanied by elevated aspartate aminotransferase (AST) and lactate dehydrogenase (LDH) levels, and succumbed to illness during the summer season. Adenoviral DNA was detected in hydrothorax fluid from patient 4 and plasma from patient 5 (Table 2). Patient 5 underwent blood cytokine and lymphocyte subset analysis, which revealed elevated cytokine levels and a marked reduction in absolute counts of lymphocyte subsets (Table 3).

**Table 2:** Laboratory findings of in five children with HAdV-55 infection in the children’s hospital of Chongqing medical university

	Case 1	Case 2	Case 3	Case 4^a^		Case 5 ^a^	
Tests time	Admission	Admission	Admission	Admission	Transfer to PICU (day 8 of hospitalization)	Admission	Transfer to PICU (day 4 of hospitalization)
Routine blood indicators (units, normal range)							
White blood cell count (*10^9^/L, 5.1–14.1)	16.18	20.08	7.59	11.58	2.82	6.96	5.06
Platelet count (*10^9^/L, 100–524)	210	256	152	478	176	304	209
Red blood cell count (*10^12^/L, 4.10–5.50)	4.85	4.43	4.68	4.68	3.47	3.53	2.99
Hemoglobin (g/L, 107–141)	116	111	128	124	89	89	79
Absolute lymphocytes (*10^9^/L, 2.40–8.70)	12.13	11.45	0.61	7.99	1.07	3.37	2.01
Absolute neutrophils (*10^9^/L, 0.80–5.80)	3.56	4.02	6.91	2.66	1.72	3.46	3
Absolute monocytes (*10^9^/L, 0.18–1.13)	0.49	1	0.08	0.69	0.03	0.13	0.05
Inflammatory indicators (units, normal range)							
C-reactive protein (CRP, mg/L, < 10)	< 8	< 8	20	< 8	< 8	14.85	23.59
Procalcitonin (PCT, ng/mL, < 0.05)	0.13	0.49	2.77	0.053	3.8	7.46	11.8
Blood biochemistry (units, normal range)							
Total protein (g/L, 56.2–72.9)	54.6	68.2	69.2	66	51.4	55.4	63.2
Albumin (g/L, 38.3–53.2)	35.4	40.2	43.4	46.5	23.4	31.6	24.1
Aspartate transferase (U/L, 29.2–54.8)	134.8	48	91.9	59	279.7	110	145
Lactate dehydrogenase (U/L, 145–353)	347.6	396	797.4	520	2778.1	1247	2392
Adenovirus load (units)							
Pharyngeal swab (copies/mL)	/	5.37*10^7^	2.19*10^5^	2.23*10^8^		1.67*10^9^	
Blood (copies/mL) 1.12*10^8^							
Pleural effusion (copies/mL)				1.60*10^5^			

^a^, case 4 and 5 included data from patients upon hospital admission and transfer to the PICU

PICU, pediatric intensive care unit

/, undetectable viral load

**Table 3:** Cytokine and lymphocyte subset results for case 5

Cytokine (units, reference range)	Results	Absolute lymphocyte count (unit, reference range)	Results
IL-2 (pg/mL, 0–9.80)	0.1	CD3 + count (cell/µL, 1 400–8 000)	730.85
IL-12p70 (pg/mL, 0–3.40)	0.2	CD3 + CD4 + count (cell/µL, 900–5 500)	509.42
IFN-α (pg/mL, 0–8.50)	508.5	CD3 + CD8 + count (cell/µL, 400–2 300)	194.8
I IL-8 (pg/mL, 0–20.60)	255.35	CD3 + CD4 + CD8 + count (cell/µL) ^a^	3.04
IL-4 (pg/mL, 0–3.00)	0.28	NK count (cell/µL, 100–1 400)	42.74
IL-5 (pg/mL, 0–3.10)	0.15	CD19 + count (cell/µL, 600–3 100)	348.96
IL-10 (pg/mL, 0–4.90)	63.69	CD45 + lymphocyte count (cell/µL) ^a^	1 128.86
TNF-α (pg/mL, 0–5.20)	1.3		
IFN-γ (pg/mL, 0–17.30)	29.83		
IL-17 A (pg/mL, 0–14.80)	0		
IL-1β (pg/mL, 0–12.4)	1		
IL-6 (pg/mL, 0–16.60)	440.98		

^a^, no established reference range

### Pulmonary imaging and fiberoptic bronchoscopy findings

**Fig. 1:**
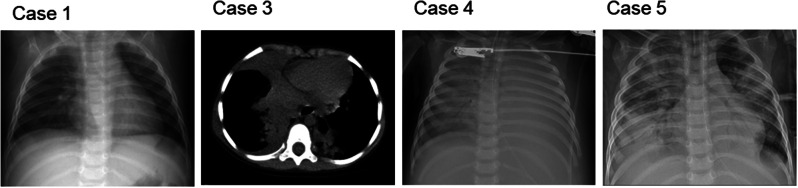
Pulmonary imaging of patients 1, 3, 4, and 5. Patient 2 underwent X-ray examinations at an external hospital and no imaging data were acquired

All patients underwent pulmonary imaging. One patient presented with imaging findings bronchiolitis, one pneumonia and three right-sided pleural effusion and bilateral pulmonary infiltrates (Fig. 1). Fiberoptic bronchoscopy and bronchoalveolar lavage were performed twice in patient 4, revealing acute tracheobronchitis. Echocardiography further identified significant cardiac anomalies, including possible partial ectopic pulmonary venous drainage (intracardiac type), atrial septal defect (type II), small left ventricle, patent ductus arteriosus, moderate tricuspid regurgitation, mild-to-moderate mitral regurgitation, and pulmonary hypertension. Imaging in patient five revealed an enlarged cardiac shadow, supported by chest ultrasound findings indicating minimal pericardial effusion.

### Treatment, outcomes and sequelae

Patients1 and 2 were treated symptomatically and subsequently discharged with favorable outcomes. Patient 3 underwent intravenous immunoglobulin (IVIG, 1 g/kg/day for two days), imipenem-cilastatin sodium (for six days) and continuous positive airway pressure (CPAP) therapy. Patient 4 was transferred to the pediatric intensive care unit (PICU) on the eighth day of hospitalization, undergoing methylprednisolone (1 g/kg every 12 h for four days), IVIG (1 g/kg for two days), cefotaxime, human albumin (25 mL every 12 h for four days), human erythrocytes (0.5 units) and invasive mechanical ventilation (IMV). Patient 5 was admitted to the PICU on the third day of hospitalization and received methylprednisolone (2 g/kg every 12 h for two days), IVIG (1 g/kg for two days), human albumin (25 mL every 12 h for two days), human erythrocytes (1 unit) and invasive mechanical ventilation (IMV). Patient 4 remained in the PICU for four days, while patient 5 succumbed to critical illness after one day in the PICU. None of the patients received extracorporeal membrane oxygenation (ECMO) or blood purification therapy (Table 1).

### Identification of HAdV-55 strain CQ2403

**Fig. 2:**
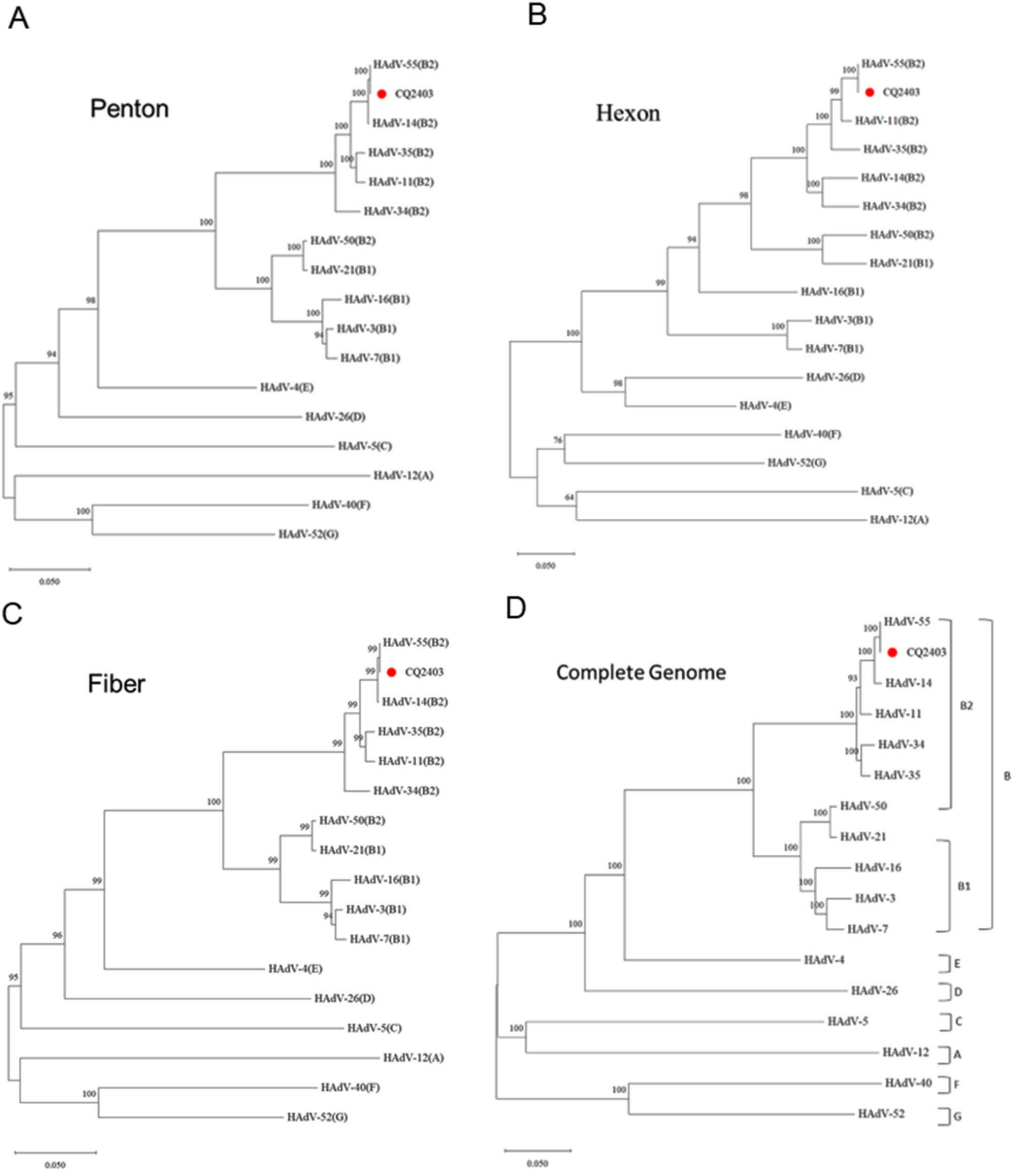
Evolution information of CQ2403 strain. (**A**-**D**) Based on the HAdV-11 QS strain, phylogenetic analysis of CQ2403 strain was performed with the entire penton gene (nt13682 to 15355), hexon gene (nt18232 to 21073), fiber gene (nt30775 to 31752) and whole-genome from 16 viral strains representing seven HAdV groups (A-G). Corresponding subgenus types are indicated in parentheses after respective serotypes. HAdV-3 (AY599834), HAdV-4 (AY594253), HAdV-5 (AY601635), HAdV-7 (AY594255), HAdV-11 (AF532578), HAdV-12 (AC_000005), HAdV-14 (AY803294), HAdV-16 (AY601636), HAdV-21 (AY601633), HAdV-26 (EF153474), HAdV-34 (AY737797), HAdV-35 (AY128640), HAdV-40 (NC_001454), HAdV-50 (AY737798), HAdV-52 (DQ923122), and HAdV-55(FJ643676). A, HAdV-A; B1, HAdV-B1; B2, HAdV-B2; C, HAdV-C; D, HAdV-D; E, HAdV-E; F, HAdV-F; G, HAdV-G. Relative phylogenetic distances were measured from the lowest scales of each phylogenetic tree (0.05)

To evaluate the potential epidemic risk associated with HAdV-55, the virus strain CQ2403 was isolated from the NPA sample of patient 5. Genomic analysis revealed that the complete genome, as well as the penton and hexon genes of CQ2403 shared 100% homology with HAdV-55. The fiber gene exhibited 99% sequence similarity, indicating minimal divergence within this region (Fig. 2). These findings highlight the relatively low mutation rates in the HAdV-55 genome, underscoring its genetic stability and potential for widespread circulation.

### Trends of clinical HAdV-55 infections worldwide

To gain a comprehensive understanding of the clinical characteristics of HAdV-55 respiratory tract infections in children, a literature review was conducted, integrating our findings with data from previously published studies. A total of 219 articles were identified, including 129 from PubMed and 90 from the China National Knowledge Infrastructure. After removing duplicates and screening titles, abstracts, and full texts, nine articles encompassing 51 cases were deemed eligible, including eight published in English [12–19] and one published in Chinese [24]. Combined with our five cases, the analysis included a total of 56 patients (Supplementary Table 1). The JBI quality assessment of included studies indicated moderate to good methodological quality (scores 4–9), with key limitations including inadequate control of confounding factors, unclear inclusion criteria in some studies, and incomplete reporting (e.g., missing demographic details in case series and unaddressed confounders in cross-sectional studies) (Supplementary Tables 2–5).

**Fig. 3:**
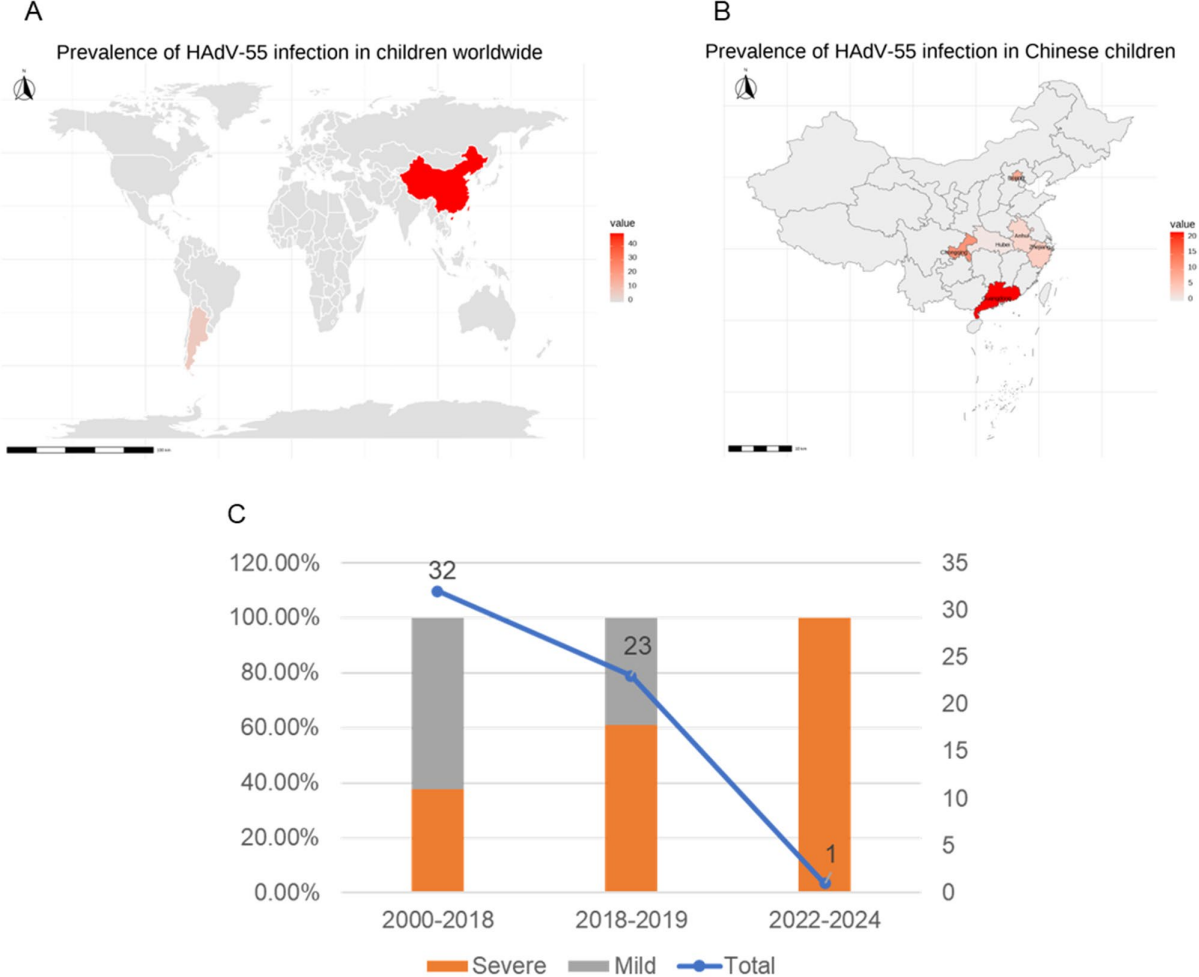
Summary of clinical HAdV-55 infections in children. Worldwide (**A**) and China (**B**) distributions of HAdV-55 in 56 children. (**C**) Temporal trends in HAdV-55 infection

Among these patients, 61.9% (26/42) were classified as severe or critical cases, while 38.1% (16/42) presented with mild infections. The overall mortality rate was 16.4% (9/55). Geographically, 12.5% (7/56) of cases originated from Buenos Aires, Argentina, while the remaining 87.5% (49/56) were from China. Within China, the distribution of cases was as follows: 39.3% (22/56) from Guangzhou, 19.6% (11/56) from Chongqing, 14.3% (8/56) from Beijing, 7.1% (4/56) from Hangzhou, 5.4% (3/56) from Anqing, and 1.8% (1/56) from Wuhan (Fig. 3A and B). To contextualize these cases within epidemiological trends, the 56 patients were categorized into three time periods: before the adenovirus outbreak (2018–2019), during the adenovirus outbreak, and after the novel coronavirus pandemic. An upward trend in severe HAdV-55 infections was observed from 2000 to 2019 (Fig. 3C). Notably, the only post-novel coronavirus case recorded in our hospital involved a critical pneumonia patient who succumbed to the infection.

Among the patients analyzed, 84.9% (45/53) were under five years of age, and 66.7% (26/39) were male. Although adenoviral infections occurred throughout the year, spring emerged as the predominant season, accounting for 44.9% (22/49) of hospitalizations. Severe or critical cases were most frequent in spring (41.2%, 7/17) and summer (35.3%, 6/17). Notably, 60% (3/5) of fatalities occurred during summer, reflecting a seasonal trend consistent with surveillance data from Osaka, Japan [25]. The most common symptoms included fever (88.9%, 16/18), cough (83.3%, 15/18), wheezing and shortness of breath (16.7%, 3/18), and sore throat and nasal congestion (5.6%, 1/18). We categorized the extractable data into two groups based on disease severity: severe pneumonia and critical pneumonia (Table 4). Additionally, we classified the patients into survival and death groups according to their outcomes (Table 5). There were no significant differences in age, gender, season, or co-infections among them and overlapped with other respiratory viral infections. However, critically ill patients had longer hospital stays and higher mortality rates (Supplementary Table 1).

**Table 4:** Clinical characteristics of HAdV-55 infection in severe and critical pediatric pneumonia patients

Variable	Critical pneumonia, *N* = 8^a^	Severe pneumonia, *N* = 9^a^	Pvalue^b^
Age (Mouths)	29 (11, 61)	16 (12, 38)	0.5
Sex			0.6
Male	5 (62%)	7 (78%)	
Female	3 (38%)	2 (22%)	
Season of occurrence		> 0.9	
Spring	3 (38%)	4 (44%)	
Autumn	2 (25%)	2 (22%)	
Summer	3 (38%)	3 (33%)	
Length of hospital stay (days)	19 (13, 26)	10 (8, 12)	0.051
Co-detection			> 0.9
No	2 (25%)	3 (33%)	
Yes	6 (75%)	6 (67%)	

^a^ Median (IQR); n (%)

^b^ Wilcoxon rank sum test; Fisher’s exact test

**Table 5:** Clinical characteristics of death and survival in children with HAdV-55 infection-induced severe pneumonia

Variable	Survival, *N* = 12^a^	Died, *N* = 5^a^	*P*-value^b^
Age (Mouths)	15 (11, 40)	45 (12, 60)	0.4
Sex			> 0.9
Male	8 (67%)	4 (80%)	
Female	4 (33%)	1 (20%)	
Season of occurrence		0.4	
Spring	6 (50%)	1 (20%)	
Autumn	3 (25%)	1 (20%)	
Summer	3 (25%)	3 (60%)	
Length of hospital stay (days)	12 (10, 19)	13 (12, 28)	0.3
Co-detection			0.6
No	3 (25%)	2 (40%)	
Yes	9 (75%)	3 (60%)	
Degree of illness			0.009
Critical pneumonia	3 (25%)	5 (100%)	
Severe pneumonia	9 (75%)	0 (0%)	

^a^ Median (IQR); n (%)

^b^ Wilcoxon rank sum test; Fisher’s exact test

**Fig. 4:**
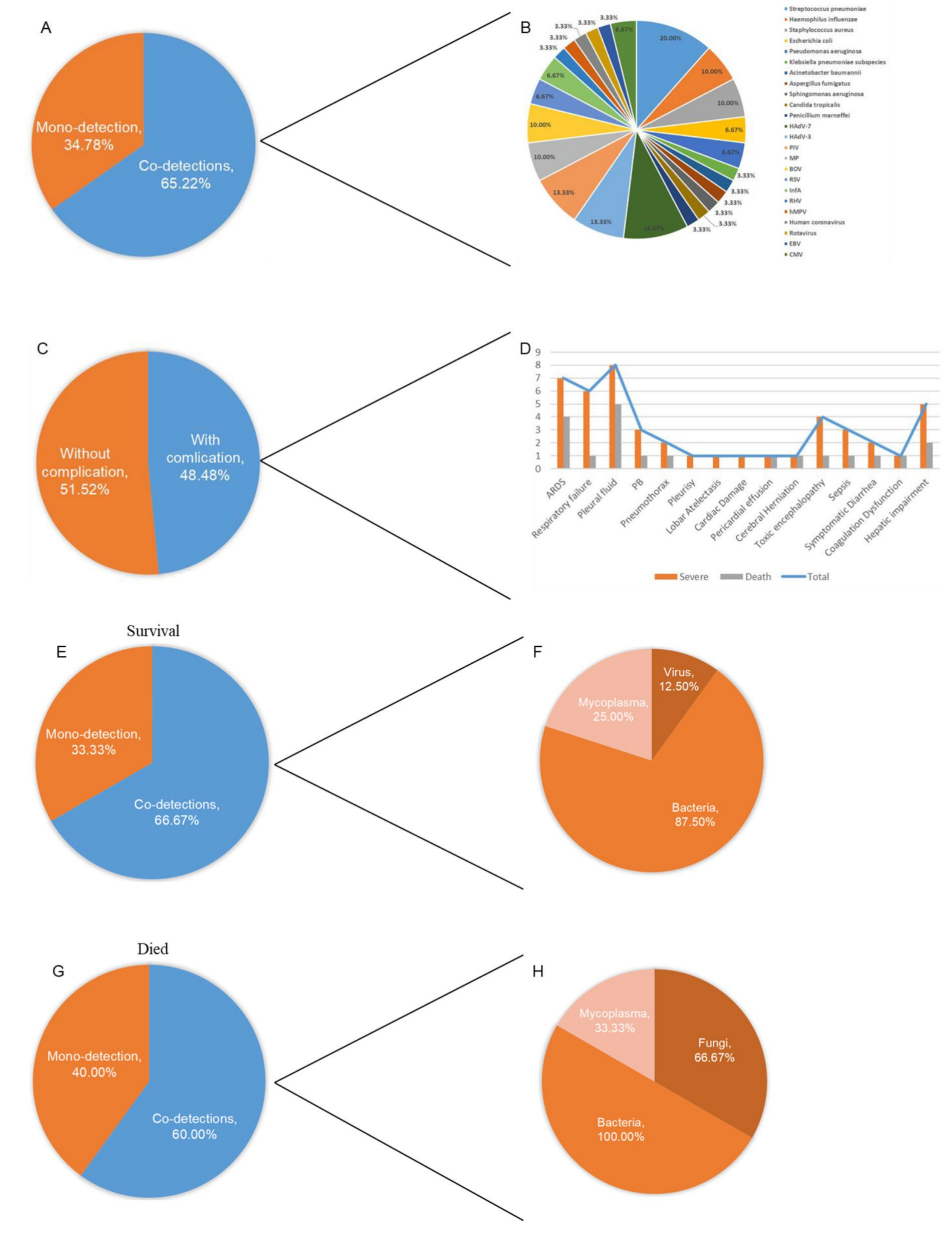
Multiple infections of clinical cases. (**A** and **B**) Mono-detection and co-detections patterns of HAdV-55 in all reported cases. (**C** and **D**) Types and frequencies of complications in children infected with HAdV-55. (**E** and **G**) Comparison of mono-detection and co-detections in relation to survival and died patients with severe HAdV-55 pneumonia. (**F** and **H**) Specific pathogens of co-detections between survival and died patients with severe HAdV-55 pneumonia. (*Suspected pathogens (such as syphilis and CMV) were not specifically enumerated.)

Lung imaging revealed infiltrates in 68.18% of patients, which appeared in all severe and critical pneumonia patients. HAdV-55 mono-detection occurred in 34.8% (16/46) of cases, while 65.2% (30/46) co-detected with other pathogens (Fig. 4A and B). Surviving children with severe pneumonia typically exhibited co-infections with bacteria, MP and viruses (Fig. 4F), those with critical pneumonia were with bacteria, MP and fungi (Fig. 4H). Notably, at least five severe or critical pneumonia cases involved mono-detected HAdV-55, including two fatalities (Table 5).

Among the patients for whom complication data were available, 51.52% (17/33) had no complications, while 48.48% (16/33) developed complications, including acute respiratory distress syndrome (ARDS, seven cases), respiratory failure (six cases), pleural effusion (eight cases), plastic bronchitis (PB, three cases), pneumothorax (two cases), pleurisy (one case), lobar atelectasis (one case), cardiac damage (one case), pericardial effusion (one case), cerebral herniation (one case), toxic encephalopathy (four cases), sepsis (three cases), symptomatic diarrhea (two cases), coagulation dysfunction (one case), and hepatic impairment (five cases) (Fig. 4C and D). Sequelae included bronchiolitis obliterans and bronchiectasis.

Among the patients, treatment approaches varied based on disease severity. IVIG was administered to 65.0% (13/20) of patients, while antibiotic therapy was used in 77.8% (14/18) of patients. IMV was required in 45.0% (9/20) of cases, methylprednisolone in 27.8% (5/18), ECMO in 16.7% (3/18), blood transfusions in 11.1% (2/18), blood purification in 11.1% (2/18), and symptomatic treatment alone in 11.1% (2/18).

## Discussion

This study provides an in-depth review of clinical data on pediatric cases of HAdV-55 infections reported to date. An upward trend in the incidence of severe HAdV-55 infections was observed from 2000 to 2019. These findings emphasize the urgent need for heightened awareness and further research into this adenoviral subtype, particularly given its association with severe illness and fatal outcomes in pediatric populations [12–19, 24].

Our findings indicated that Guangzhou reported the highest incidence of HAdV-55 infections, followed by Chongqing. Supporting this, previous research has identified HAdV-55 as an increasingly common cause of life-threatening pneumonia in Guangzhou [17]. These results highlight the importance of heightened surveillance for HAdV-55, particularly given its role as a significant pathogen in respiratory infections associated with severe morbidity and mortality.

The primary clinical symptoms of HAdV-55 infection, including fever and cough, were comparable to those of other respiratory viral infections. However, critical infections patients experienced prolonged symptoms, indicating a more aggressive disease course [13, 16]. Patients 4 and 5 demonstrated a rapid and marked decline in whole blood cell counts, hemoglobin levels, and albumin levels within a short period, accompanied by significant elevations in AST, LDH, and PCT, well-recognized indicators of disease severity [26, 27]. The elevation of cytokine profile was akin to those observed in adults with HAdV-55 [28]. While, the declination of lymphocyte counts suggested rapid immune depletion and the development of a cytokine storm. Such immune dysregulation can compromise the integrity of the pulmonary microvasculature and alveolar epithelial barriers, resulting in alveolar edema, hypoxia, and potentially ARDS [29]. However, the precise mechanisms underlying HAdV-55-induced immune dysregulation and immunosuppression remain poorly understood and warrant further investigation through experimental studies.

Previous studies have shown that critically ill patients exhibit significantly higher levels of PCT, CRP, and lactate dehydrogenase (LDH), whereas patients with severe pneumonia have significantly lower albumin levels [10]. But little known for the long-term information, continuous monitoring should be taken for prognosis to track the changes of the HAdV-55 infected patients. HAdV-55 infections can cause severe acute lung injury and are frequently associated with secondary infections (Fig. 4A and B). Notably, at least five severe or critical pneumonia cases involved mono-detected HAdV-55, including two fatalities (Table 5), indicating that HAdV-55 can still be highly pathogenic in the absence of co-infections. This virulence may be attributed to the immune evasion strategies of the virus, including inhibition of programmed apoptosis in infected cells and suppression of the host DNA damage response [30, 31]. Pleural effusion and bilateral pulmonary infiltrates constitute primary radiographic findings in severe HAdV-55 infections, mirroring presentations seen in bacterial or mycoplasma pneumonias. These indicated us diagnosis of HAdV infection should be considered in patients with severe pneumonia with negative bacterial cultures and failure to respond to antibiotics [10, 32]. Adenoviral load is strongly correlated with increased disease severity [33]. Furthermore, we suggest monitoring sterile body fluids such as blood, urine, pleural fluid, and cerebrospinal fluid to ascertain viral dissemination and assess disease progression. HAdV-55 is associated with severe infections characterized by extensive intrapulmonary damage and extrapulmonary complications affecting the cardiovascular, hepatic, gastrointestinal, neurological, and hematological systems. These manifestations reflect breaches in the alveolar-capillary barrier, driven by excessive inflammatory responses.

Previous studies have indicated that therapeutic interventions, such as IVIG and advanced respiratory support (e.g., ECMO), may improve outcomes and reduce mortality in critically ill children with HAdV-7 infection [32]. However, in our study, despite aggressive interventions, there were nine patients died [13, 16]. This poor outcome may be partially attributed to the variable titers of specific adenovirus antibodies present in commercially available IVIG preparations [34]. To address this limitation, the development of passive immunotherapy using hyperimmune IVIG derived from plasma donated by individuals recovering from HAdV-55 infections offers a promising alternative for managing patients with severe HAdV-55 infection [35]. Furthermore, all complications and sequelae were observed exclusively in severe or critical pneumonia patients, underscoring the severe systemic impact of HAdV-55 infection in this subgroup.

This study has several limitations. Firstly, the included studies are all retrospective designs, with some being mixed studies combining clinical and basic research. Issues such as insufficient control of confounding factors, unclear inclusion criteria, and incomplete reporting (e.g., case series lacking demographic details, and cross-sectional studies not addressing confounding factors) exist. Secondly, the 56 cases of pediatric HAdV-55 infection data come only from China and Argentina, and the geographic distribution of the sample may introduce regional bias when understanding its global distribution. Thirdly, standardizing clinical data from non-Chongqing regions is difficult, which limits a comprehensive assessment of the clinical characteristics of the cases. Fourthly, mixed infections are common, making it challenging to determine whether the severity of the disease is solely caused by HAdV-55. Finally, the sample size in our study is limited, especially for severe/death cases, and there is insufficient immune monitoring for severe cases (e.g., case 4 did not test lymphocyte subsets), which hinders the analysis of immune characteristics in severe infections. Future studies should conduct multi-center and multi-region epidemiological research, establish standardized data collection protocols to improve analysis efficiency, design experiments to control for mixed infection variables to clarify the independent pathogenicity of HAdV-55, and strengthen immune monitoring and epidemiological data collection for severe cases.

## Conclusions

The genetic inheritance of HAdV-55 remained stable, with an upward trend of HAdV-55 severe infection among children from 2000 to 2019. Early clinical symptoms of HAdV-55 infection were nonspecific and overlapped with those of other respiratory viral infections. Despite being susceptible to co-detect with other pathogens, HAdV-55 also had the capacity to cause severe illness independently. Rapid declines in blood cell counts and serum albumin, along with dynamic monitoring of viral loads in sterile fluids, may serve as prognostic indicators. Our study describes the clinical characteristics of pediatric respiratory HAdV-55 infections, offering limited evidence for the identification and management of severe cases. This research provides new insights for future studies.

## Electronic supplementary material

Below is the link to the electronic supplementary material.

Supplementary Material 1

Supplementary Material 2

## Data Availability

The datasets generated and/or analyzed during the current study are available in the [Sequence Read Archive (SRA)] repository, [https://www.ncbi.nlm.nih.gov/sra/PRJNA1241473].Sequence data that support the findings of this study have been deposited in the NCBI database under GenBank accession numbers PV368459.

## Abbreviations

HAdV: 55-Human adenovirus type 55
HAdV: Human adenovirus
ARDS: acute respiratory distress syndrome
NPAs: Nasopharyngeal aspirates
CRP: C-reactive protein
AST: aspartate aminotransferase
PCT: procalcitonin
IFN: interferon
NK: natural killer
EBV: Epstein-Barr virus
CMV: cytomegalovirus
IVIG: intravenous immunoglobulin
PICU: pediatric intensive care unit
CPAP: continuous positive airway pressure
IMV: invasive mechanical ventilation
ECOM: membrane oxygenation
LDH: lactate dehydrogenase
PB: plastic bronchitis
