# Correction: Clinical features of HAdV-55 in children with respiratory tract infections: a retrospective case series and literature review

**DOI:** 10.1186/s12879-025-11060-9

**Published:** 2025-05-06

**Authors:** Lifen Rao, Yueqiang Fu, Ying Lu, Jianhua Wei, Zhongying Yang, Mengling Qi, Chengjun Liu, Yushun Wan, Enmei Liu, Na Zang

**Affiliations:** 1 Department of Respiratory, National Clinical Research Center for Child Health and Disorders, Ministry of Education Key Laboratory of Child Development and Disorders. China International Science and Technology Cooperation base of Child development and Critical Disorders, Chongqing Key Laboratory of Child Rare Diseases in Infection and Immunity, Children’s Hospital of Chongqing Medical University, Chongqing, 400014 China; 2 Department of Pediatric Intensive Care Unit, Children’s Hospital of Chongging Medical University, Chongqing, 400014 China; 3 College of Basic Medicine, Chongqing Medical University, Chongqing, 400016 China

Following publication of the original article [1], we were notified that the first two authors have made equal contributions and should be marked as co-first authors.

The original article has been corrected and the note was added: “† Lifen Rao and Yueqiang Fu are co-first authors and contributed equally to this work.”

## References

[1] Rao, et al. BMC Infect Dis. 2025;25:553. 10.1186/s12879-025-10890-x. 40247166 10.1186/s12879-025-10890-x PMC12007208

