# Correction: Clinical features of HAdV-55 in children with respiratory tract infections: a retrospective case series and literature review

**DOI:** 10.1186/s12879-025-12290-7

**Published:** 2025-12-23

**Authors:** Lifen Rao, Yueqiang Fu, Ying Lu, Jianhua Wei, Zhongying Yang, Mengling Qi, Chengjun Liu, Yushun Wan, Enmei Liu, Na Zang

**Affiliations:** 1 Department of Respiratory Children’s Hospital of Chongqing Medical University, National Clinical Research Center for Child Health and Disorders, Ministry of Education Key Laboratory of Child Development and Disorders, China International Science and Technology Cooperation base of Child development and Critical Disorders, Chongqing Key Laboratory of Child Rare Diseases in Infection and Immunity, Chongqing, 400014 China; 2 Department of Pediatric Intensive Care Unit, Children’s Hospital of Chongging Medical University, Chongqing, 400014 China; 3 College of Basic Medicine, Chongqing Medical University, Chongqing, 400016 China

**Correction: BMC Infect Dis (2025) 25:553**

**https://doi.org/10.1186/s12879-025-10890-x**

Following publication of the original article [1], we were notified of a few errors in Table 1; Fig. 4:

Table 1: The “Admission time” for Case 5 was displayed incorrectly due to character encoding issues, resulting in the failure to display the specific date. The correct admission time should be [2024/3/11] instead of 45,363, as originally published.

Figure 4A: The labels for " Mono-detection” and “Co-infections” were inadvertently reversed. According to the data in Supplementary Table 1, the correct values are: Mono-detection (16/46) and Co-infections (30/46).

Figure 4G: The statistical data on infection types among deceased patients with severe pneumonia was inaccurate. Based on Supplementary Table 1, the correct breakdown is: 3 cases of mixed infection and 2 cases of single infection.

Figure 4F and H: These two subfigures contained statistical errors and incorrect use of denominators. The denominator should be uniformly defined as the total number of patients with mixed infection. The authors have recalculated the data in accordance with Supplementary Tables 1, and the revisions are reflected in the new Fig. 4. It should be noted that *suspected pathogens (such as syphilis and CMV) were not specifically enumerated*. We would like to clarify that although the figures contained errors, the corresponding textual descriptions in the article do not elaborate on these specific data points, so no revisions to the main text are required.

Originally published Fig. 4:

**Figure:**
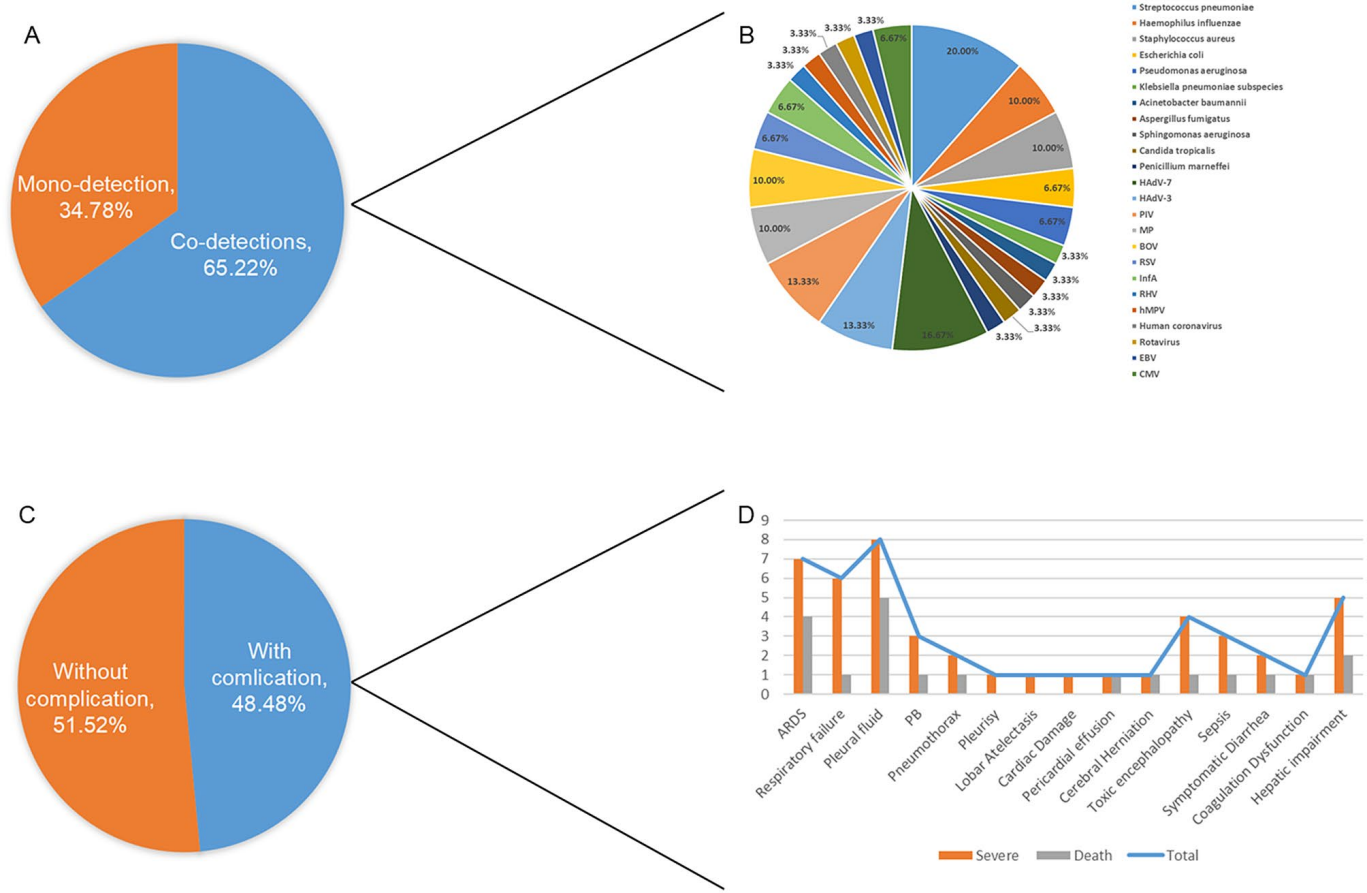

Corrected Fig. 4:

**Figure:**
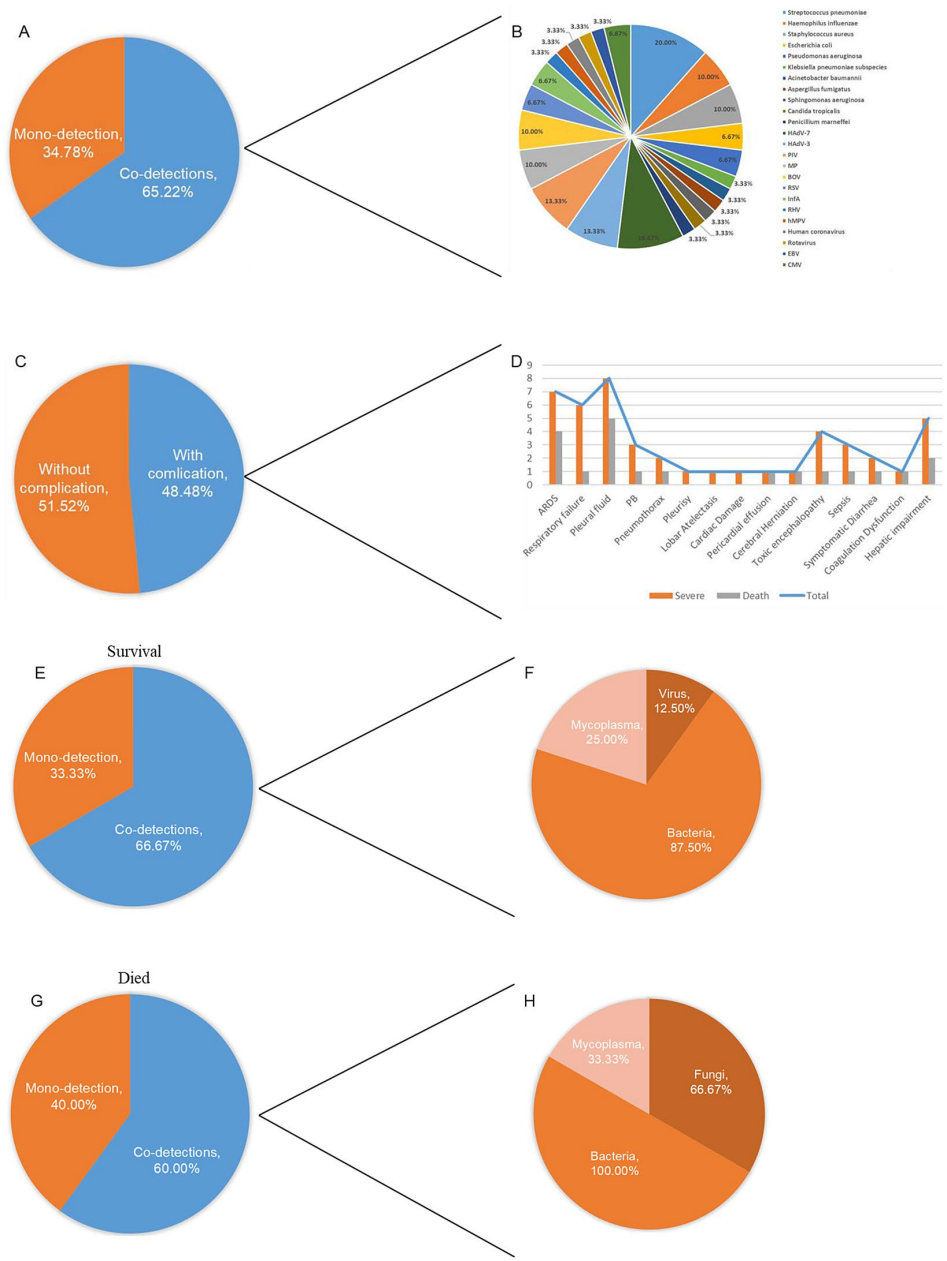

The original article has been corrected.
