# Supplementary material for: Clinical features of HAdV-55 in children with respiratory tract infections: a retrospective case series and literature review
Source: BMC Infect Dis. 2025 Apr 17;25:553. doi: 10.1186/s12879-025-10890-x (PMC12007208; doi:10.1186/s12879-025-10890-x)
Supplement: Supplementary file 1 — Supplementary Material 1 [file 12879_2025_10890_MOESM1_ESM.pdf]

SUPPLEMENTARY TABLE 1 Clinical data of 56 children with HAdV-55 infection

Cases	Age range (years)	City, country	Sex	Admission time (season)	Primary diagnosis	Co-infection	Complications	Chief complaint	Length of hospital stay (days)	Treatment	Outcome	Sequelae
Case 1	≤5	Chongqing, China	Male	Autumn	Severe pneumonia	*Staphylococcus aureus*	Respiratory failure, symptomatic diarrhea, hepatic impairment	Fever 3 days, cough 2 days, wheezing, shortness of breath 1 day	3	Symptomatic supportive therapy	Survival	None
Case 2	≤5	Chongqing, China	Male	Summer	Bronchitis	EBV, CMV	None	Fever 11 days	5	Symptomatic supportive therapy	Survival	None
Case 3	≤5	Chongqing, China	Male	Summer	Severe pneumonia	*Streptococcus pneumoniae, Haemophilus influenzae*	Septicemia, respiratory failure, pleural effusion, pleurisy, hepatic impairment	Cough 1 month, aggravated with fever 5 days	9	IVIG, Imipenem, and CPAP	Survival	None

Case 4	≤5	Chongqing, China	Female	Summer	Critical pneumonia	*Streptococcus pneumoniae, syphilis suspected*	Respiratory failure, brain herniation, pleural effusion, symptomatic diarrhea, coagulation disorders, hepatic impairment, toxic encephalopathy? Intracranial hemorrhage? Cerebral infarction?	Cough more than 1 month, aggravated with intermittent fever 1 week	12	Methylprednisolone, IVIG, human albumin[a], erythrocyte suspension[b], cefazoxime, and IMV	Died
Case 5	≤5	Chongqing, China	Male	Spring	Critical pneumonia	None	ARDS, sepsis, pleural effusion, hepatic impairment	Cough more than 1 month with fever 6 days	4	Methylprednisolone, IVIG, human albumin[a], erythrocyte suspension[b], cefoperazone sulbactam sodium, and IMV	Died

Case 6	> 5	Chongqing, China	Male	Summer	Upper respiratory tract infection	*streptococcus pneumoniae*	None	Fever, sore throat, nasal congestion 8 days	3	Amoxicillin sodium sulbactam sodium	Survival	None
Case 7	≤5	Chongqing, China	Male	Spring	Delayed pneumonia	*Streptococcus pneumoniae,* PIV, *Haemophilus influenzae*	Toxic encephalopathy	Recurrent coughing and wheezing 1 month	11	Cefamandole	Survival	None
Case 8	≤5	Chongqing, China	Male	Spring	Interstitial pneumonia	BOV *Staphylococcus aureus*	/	/	/	/	Survival	None
Case 9	≤5	Chongqing, China	Male	Spring	Severe pneumonia	CMV suspect, *Klebsiella pneumoniae subspecies*	Respiratory failure, toxic encephalopathy	Cough with fever 10 days	10	Methylprednisolone, cefamandole, piperacillin tazobactam sodium, cefoxitin sodium, and clindamycin	Survival	None
Case 10	≤5	Chongqing, China	Male	Spring	Severe pneumonia	None	Toxic encephalopathy	Cough with fever 6 days	6	Cefoxitin sodium, and piperacillin tazobactamna	Survival	None

Case 11	≤5	Chongqing, China	Male	Winter	Bronchitis	None	None	/	/	None	Survival	None
Case 12	≤5	Guangzhou, China	Male	Autumn	Critical pneumonia	MP, *E. coli*	ARDS, pneumothorax, pleural effusion	Cough with wheezing 12 days, fever 8 days	25	IVIG, piperacillin sulbactam sodium, and IMV	Survival	BO
Case 13	≤5	Guangzhou, China	Male	Summer	Critical pneumonia	MP, a*cinetobacter baumannii, pseudomonas aeruginosa, aspergillus fumigatus*	ARDS, pneumothorax, pleural effusion, PB	Recurrent fever and cough for more than 20 days, shortness of breath 4 days	28	Methylprednisolone, IVIG, erythromycin, meropenem, linezolid, voriconazole, and IMV	Died	
Case 14	> 5	Guangzhou, China	Male	Summer	Critical pneumonia	None	ARDS, pleural effusion	Recurrent fever 14 days, cough 7 days	13	IVIG, imipenem, meropenem, vancomycin and IMV, ECOM, and blood purification	Died	

Case 15	≤5	Guangzhou, China	Male	Autumn	Critical pneumonia	*Sphingomonas aeruginosa, candida tropicalis, penicillium marneffei*	ARDS, pleural effusion, PB	Recurrent fever 15 days	45	Piperacillin tazobactam, voriconazole, caspofungin and IMV, ECOM, and blood purification	Died	
Case 16	> 5	Guangzhou, China	Female	Spring	Critical pneumonia	*Methicillin-resistant staphylococcus aureus*	ARDS, pleural effusion, PB	Recurrent fever more than 20 days, cough 10 days	25	IVIG, cefoperazone sulbactam, vancomycin and MIV, and ECOM	Survival	None
Case 17	≤5	Guangzhou, China	Female	Spring	Critical pneumonia	Rotavirus	ARDS	Recurrent cough and shortness of breath 15 days	13	IVIG, cefoperazone sulbactam, and MIV	Survival	None
Case 18	≤5	Guangzhou, China	Male	Spring	Severe pneumonia	None	None	Recurrent cough more than 2 months, fever 5 days	12	IVIG	Survival	BO

Case 19	≤5	Guangzhou, China	Female	Summer	Severe pneumonia	MP	None	Cough more than 20 days, fever 6 days	12	IVIG, and azithromycin	Survival	None
Case 20	≤5	Guangzhou, China	Male	Summer	Severe pneumonia	None	None	Cough 1 month, fever 2 days	26	Methylprednisolone, and IVIG	Survival	BO
Case 21-28	≤5	Bejing, China	5Male3Female	Seven in spring, one in autumn	Two severe pneumonia and six mild pneumonia or respiratory tract infection	Two mono-infection and six co-infections (three with PIV, two with *streptococcus pneumoniae* and one with RHV, InfA, human coronavirus, BOV, hMPV, *E. coli,* and *Haemophilus influenzae*)	Two with respiratory failure, one with cardiac damage and one with hepatic damage	/	11.33 ± 6.86	One MIV（1/6）, two IVIG（2/7）	Survival	None

Case 29	≤5	Buenos Aires, Argentina	Female	Autumn	Severe capillary bronchitis	*Pseudomonas aeruginosa*	Sepsis, pulmonary atelectasis	/	/	/	Survival	Left lower lobe bronchiectasis
Case 30	> 5	Buenos Aires, Argentina	Male	Winter	/	None	None	/	/	/	Survival	None
Case 31	> 5	Buenos Aires, Argentina	Female	Winter	Tonsillitis	None	None	/	/	/	Survival	None
Case 32	≤5	Buenos Aires, Argentina	Male	Spring	Capillary bronchitis	None	None	/	/	/	Survival	None
Case 33	≤5	Buenos Aires, Argentina	Female	Spring	Capillary bronchitis	None	None	/	/	/	Survival	None
Case 34	> 5	Buenos Aires, Argentina	Male	Autumn	Upper respiratory tract infection	None	None	/	/	/	Survival	None
Case 35	≤5	Buenos Aires,	Female	Autumn	Tonsillitis	None	None	/	/	/	Survival	None

		Argentina										
Case 36-46	nine less than 5, two more than 5	Guangzhou, China	/	Two in spring, three in summer, three in autumn and three in winter	One with severe pneumonia, remains unknown	Two single detection and nine co-detections (four with HAdV-3, five with HAdV-7, one with InfA, one with Human BOV, and two with RSV)	/	/	/	/	One died and ten survived	/
Case 47-48	/	Guangzhou, China	/	/	Two with severe pneumonia	/	/	/	/	/	One died and two survived	/
Case 49-52	≤5	Hangzhou, China	/	/	Four with severe pneumonia	/	/	/	/	/	Two died and two s Survival	/
Case 53	≤5	Anqing, China	Female	Spring	/	/	/	/	/	/	Survival	/
Case	≤5	Anqing,	Male	Spring	/	/	/	/	/	/	Survival	/

54		China										
Case 55	≤5	Anqing, China	Male	Spring	/	/	/	/	/	/	Survival	/
Case 56	/	Hubei, China	Female	/	/	/	Sepsis	/	/	/	/	/

Annotation, patients, cases 1-11, admitted to the Children’s Hospital of Chongqing Medical University. Cases 1-5 were reported by our research team, cases 6-11 were derived from existing literature.

EBV, Epstein-barr virus. CMV, cytomegalovirus. MP, mycoplasma pneumoniae. E. coli, escherichia coli. RSV, respiratory syncytial virus. hMPV, human metapneumovirus. InfA, Influenza A virus. RHV, rhinovirus.

BOV, bocavirus. PIV, parainfluenza virus. PB, plastic bronchitis. BO, bronchiolitis obliterans.

/, data cannot be extracted.

[a],Human albumin was administered to correct hypoalbuminemia.

[b], erythrocyte suspension was utilized to manage anemia.
