# Supplementary material for: Clinical features of HAdV-55 in children with respiratory tract infections: a retrospective case series and literature review
Source: BMC Infect Dis. 2025 Apr 17;25:553. doi: 10.1186/s12879-025-10890-x (PMC12007208; doi:10.1186/s12879-025-10890-x)
Supplement: Supplementary file 2 — Supplementary Material 2 [file 12879_2025_10890_MOESM2_ESM.pdf]

SUPPLEMENTARY TABLE 2 Risk of bias assessment of included case control studies.

Serial	Author	Year	Were the groups comparable other than the presence of disease in cases or the absence of disease in controls?	Were cases and controls matched appropriately?	Were the same criteria used for identification of cases and controls?	Was exposure measured in a standard, valid and reliable way?	Was exposure measured in the same way for cases and controls?	Were confounding factors identified?	Were strategies to deal with confounding factors stated?	Were outcomes assessed in a standard, valid and reliable way for cases and controls?	Was the exposure period of interest long enough to be meaningful?	Was appropriate statistical analysis used?	Score
1	Lili Xu	2018	Yes	Yes	Yes	Yes	Yes	Unclear	No	Yes	No	Yes	7

SUPPLEMENTARY TABLE 3 Risk of bias assessment of included case reports

Serial	Author	Year	Were patient's demographic characteristics clearly described?	Was the patient's history clearly described and presented as a timeline?	Was the current clinical condition of the patient on presentation clearly described?	Were diagnostic tests or assessment methods and the results clearly described?	Was the intervention(s) or treatment procedure(s) clearly described?	Was the post-intervention clinical condition clearly described?	Were adverse events (harms) or unanticipated events identified and described?	Does the case report provide takeaway lessons?	Score

1	Shuping Jing	2019	Yes	Yes	Yes	Yes	Yes	Yes	No	Yes	7

SUPPLEMENTARY TABLE 4 Risk of bias assessment of included case series

Serial	Author	Year	Were there clear criteria for inclusion in the case series?	Was the condition measured in a standard, reliable way for all participants included in the case series?	Were valid methods used for identification of the condition for all participants included in the case series?	Did the case series have consecutive inclusion of participants?	Did the case series have complete inclusion of participants?	Was there clear reporting of the demographics of the participants in the study?	Was there clear reporting of clinical information of the participants?	Were the outcomes or follow up results of cases clearly reported?	Was there clear reporting of the presenting site(s)/clinic(s) demographic information?	Was statistical analysis appropriate?	Score
1	Dongwei Zhang	2022	Yes	Yes	Yes	Yes	Yes	Yes	Yes	Yes	Unclear	Yes	8
2	Qing-Bin Lu	2014	Yes	Yes	Yes	Yes	Yes	Yes	Yes	Yes	Unclear	Yes	9
3	Adriana E. Kajon	2013	Yes	Yes	Yes	Yes	Yes	Yes	No	Yes	Unclear	Yes	8

SUPPLEMENTARY TABLE 5 Risk of bias assessment of included analytical cross-sectional studies

Serial	Author	Year	Were the criteria for inclusion in the sample clearly defined?	Were the study subjects and the setting described in detail?	Was the exposure measured in a valid and reliable way?	Were objective, standard criteria used for measurement of the condition?	Were confounding factors identified?	Were strategies to deal with confounding factors stated?	Were the outcomes measured in a valid and reliable way?	Was appropriate statistical analysis used?	Score
1	Caiyun Wang	2021	Yes	Yes	Yes	Yes	Unclear	No	Yes	Yes	6
2	Yi Chen	2022	Yes	Yes	Yes	Yes	Unclear	No	Yes	Yes	6
3	Shi-ying Chen	2020	No	Yes	Yes	Yes	No	No	Yes	Yes	5
4	Jianing Li	2021	No	Yes	Yes	Unclear	No	No	Yes	Yes	4
